# Isolation of Nontuberculous Mycobacteria in Southeast Asian and African Human Immunodeficiency Virus–infected Children With Suspected Tuberculosis

**DOI:** 10.1093/cid/ciy897

**Published:** 2019-01-28

**Authors:** Laurence Borand, Agathe de Lauzanne, Ngoc Lan Nguyen, Sokleaph Cheng, Thu Hang Pham, Sara Eyangoh, Abdoul-Salam Ouedraogo, Vibol Ung, Philippe Msellati, Mathurin Tejiokem, Boubacar Nacro, Malin Inghammar, Bunnet Dim, Christophe Delacourt, Sylvain Godreuil, Stéphane Blanche, Olivier Marcy, Pediatric Asian African Network for Tuberculosis and HIV Research (PAANTHER) Study Group

**Affiliations:** 1 Epidemiology and Public Health Unit, Institut Pasteur du Cambodge, Phnom Penh, Cambodia; 2 Biochemistry, Hematology and Immunology Department, Pham Ngoc Thach Hospital, Ho Chi Minh City, Vietnam; 3 Medical Biology Unit, Institut Pasteur du Cambodge, Phnom Penh, Cambodia; 4 Microbiology Department, Pham Ngoc Thach Hospital, Ho Chi Minh City, Vietnam; 5 Service de Mycobactériologie, Centre Pasteur du Cameroun, Réseau International des Instituts Pasteur, Yaounde, Cameroon; 6 Centre Hospitalier Universitaire Souro Sanou, Bobo Dioulasso, Burkina Faso; 7 TB/HIV Department, National Pediatric Hospital, Phnom Penh, Cambodia; 8 University of Health Sciences, Phnom Penh, Cambodia; 9 UMI 233-TransVIHMI, Institut de Recherche pour le Développement (IRD), U1175, Université de Montpellier, France; 10 Service d’Epidémiologie et de Santé Publique, Centre Pasteur du Cameroun, Réseau International des Instituts Pasteur, Yaounde, Cameroon; 11 Service de Pédiatrie, Centre Hospitalier Universitaire Souro Sanou, Bobo Dioulasso, Burkina Faso; 12 Department of Clinical Sciences LUND, Section for Infection Medicine, Lund University, Sweden; 13 Service de Pneumologie et d’Allergologie Pédiatriques, Hôpital Necker-Enfants Malades, Assistance Publique–Hôpitaux de Paris (AP-HP), France; 14 Laboratoire de Bactériologie, Centre Hospitalier Universitaire de Montpellier, France; 15 France (SG) Unité Mixte de Recherche (UMR) Maladies Infectieuses et Vecteurs : écologie, génétique, évolution et contrôle (MIVEGEC) IRD - Centre National de la Recherche Scientifique (CNRS), Université de Montpellier, IRD, délégation Occitanie, Montpellier, France (SG); 16 Unité d’Immunologie Hématologie Rhumatologie Pédiatrique, Hôpital Necker Enfants Malades, AP-HP, Paris, France; 17 Université de Bordeaux, Centre Institut national de la santé et de la recherche médicale U1219, Bordeaux Population Health, France

**Keywords:** HIV, children, nontuberculous mycobacteria, tuberculosis

## Abstract

We enrolled 427 human immunodeficiency virus–infected children (median age, 7.3 years), 59.2% severely immunodeficient, with suspected tuberculosis in Southeast Asian and African settings. Nontuberculous mycobacteria were isolated in 46 children (10.8%); 45.7% of isolates were *Mycobacterium avium* complex. Southeast Asian origin, age 5–9 years, and severe immunodeficiency were independently associated with nontuberculous mycobacteria isolation.

**Clinical Trials Registration:**

NCT01331811.

Nontuberculous mycobacteria (NTM) are saprophytic organisms present in the environment [1]. NTM are not systematically pathogenic but can cause various diseases in humans including respiratory disease, lymphadenitis, skin and soft tissue infection, and disseminated disease occurring mostly in immunodeficient hosts [2].

The diagnosis of NTM pulmonary disease is challenging in settings of high tuberculosis (TB) burden as NTM can also be detected by smear microscopy, and clinical and radiological features may mimic TB. However, unlike *Mycobacterium tuberculosis* (MTB), for which any positive culture is interpreted as TB disease, the major challenge with diagnosis and management of NTM is to differentiate environmental contamination, or colonization, from disease [3]. Criteria to help differentiate colonization from respiratory disease have been proposed by the American Thoracic Society (ATS) for adults, but these are not validated in children [2].

Human immunodeficiency virus (HIV)–infected adults and children are particularly at risk of NTM-related diseases, including respiratory and disseminated infections due to the *Mycobacterium avium* complex (MAC) [3]. NTM infections, MAC in particular, are globally occurring in adults and children with profound immunodeficiency and CD4 count usually <50 cells/µL. Data regarding epidemiology and clinical significance of NTM isolation in HIV-infected children from high-TB-burden and resource-limited countries remain scarce [1, 4, 5].

We assessed the rate of NTM isolation in HIV-infected children with a clinical suspicion of TB in a prospective cohort followed in 4 countries with high and very high TB burden in Southeast Asia and Africa, as well as NTM species distribution and factors associated with NTM isolation in this cohort.

## METHODS

The French National Agency for Research on AIDS and Viral Hepatitis (ANRS) 12229 Pediatric Asian African Network for Tuberculosis and HIV Research (PAANTHER) 01 study (ClinicalTrials.gov identifier NCT01331811) was a TB diagnostic cohort study that enrolled HIV-infected children aged ≤13 years with suspected intrathoracic TB from 8 hospitals in Burkina Faso, Cambodia, Cameroon, and Vietnam, from April 2010 to May 2014. Relevant ethics committees, institutional review boards, and health authorities in each country approved the study. We included in this analysis children who had at least one interpretable culture result on any microbiological sample.

Inclusion procedures and study design were described elsewhere [6]. According to his/her age, each child had 5 or 6 systematic bacteriological samples, including self-expectorated sputum or gastric aspirate, nasopharyngeal aspirate, string test, and stools collected. All samples were tested with smear microscopy, Xpert MTB/RIF assay (Xpert, Cepheid, Sunnyvale, California, USA), mycobacterial culture on Lowenstein-Jensen medium, and automated liquid culture (Bactec MGIT, Becton Dickinson, Maryland, USA), except in Burkina Faso where culture was only performed on solid media. MTB complex was identified by biochemical method, immunochromatographic tests, or commercial multiplex line-probe assays. Speciation of NTM was performed using the GenoType Mycobacterium CM/AS line probe assay (Hain Lifescience, Nehren, Germany) in Cambodia and Cameroon, and using an in-house 16S ribosomal RNA sequencing in Vietnam. NTM treatment was initiated on a case-by-case basis based on lack of response to prior TB treatment, species of pathogen identified, and immune status. Children were followed for 6 months after enrollment.

We used χ^2^ or Fisher exact tests, and Student or Wilcoxon rank-sum tests to compare characteristics between patients with and without NTM isolated. Factors associated with NTM isolation overall were evaluated using logistic regression models. We entered in multivariate models previously described associated characteristics and variables associated with the outcome in univariate analysis at a threshold of *P* <.2. We obtained final models using manual stepwise backward selection. We performed analyses using Stata software, version 13.1 (StataCorp, College Station, Texas, USA). All tests were two-sided and a *P* value <.05 was considered statistically significant.

## RESULTS

Of 438 HIV-infected children enrolled in the study, 5 died and 5 withdrew before sample collection, and one had a positive culture without identification. Finally, we included 427 children in this analysis (Supplementary Figure 1), including 245 (59.2%) with severe immunodeficiency (Supplementary Table 1).

NTM were isolated in 46 (10.8%) children, including 5 (1.2%) with both NTM and MTB. Additionally, 50 (11.7%) children had bacteriologically confirmed MTB alone. NTM were isolated in 16.7% of Southeast Asian children and 2.7% of African children.

NTM were isolated from 102 specimens. Among these, species identification was successful for 85 (83.3%) specimens obtained from 41 of 46 (89.1%) children. Overall, 11 different NTM species were identified (Table 1). The most frequently isolated NTM species were MAC in 21 (45.7%) children, *Mycobacterium fortuitum*, *Mycobacterium simiae*, and *Mycobacterium scrofulaceum*. The same NTM species was identified in at least 2 separate specimens in 18 (39.1%) children. MAC was isolated almost exclusively in Southeast Asian children (20 of 21 children).

**Table 1.** Nontuberculous Species Identified

	All Children With NTM Identified^a^ (n = 46)			Severe Immunodeficiency (n = 33)			Absence of Severe Immunodeficiency (n = 13)		
Mycobacterial Species	No.	(%)	Identical Species in ≥2 Samples, No.	No.	(%)	Identical Species in ≥2 Samples, No.	No.	(%)	Identical Species in ≥2 Samples, No.
1 NTM species identified	28	(60.9)	13	18	(39.1)	12	10	(21.7)	1
* *MAC	13	(28.3)	9	10	(21.7)	9	3	(6.5)	
* M. fortuitum*	7	(15.2)	1	3	(6.5)		4	(8.7)	1
* M. simiae*	2	(4.3)	1	2	(4.3)	1			
* M. kansasii*	1	(2.2)	1	1	(2.2)	1			
* M. scrofulaceum*	2	(4.3)	1	1	(2.2)	1	1	(2.2)	
* M lentiflavum*	1	(2.2)		1	(2.2)				
* M. gordonae*	1	(2.2)					1	(2.2)	
* M. interjectum*	1	(2.2)					1	(2.2)	
2 NTM species identified	11	(23.9)	3	9	(19.6)	3	2	(4.3)	0
* *MAC + nontypeable NTM^b^	3	(6.5)	2^c^	3	(6.5)	2^c^			
* *MAC + *M. simiae*	2	(4.3)		2	(4.3)				
* *MAC + *M. scrofulaceum*	1	(2.2)	1^d^	1	(2.2)	1			
* *MAC + unidentified NTM^e^	1	(2.2)		1	(2.2)				
* M. fortuitum* + *M. interjectum*	1	(2.2)					1	(2.2)	
* M. gordonae* + nontypeable NTM	1	(2.2)					1	(2.2)	
* M. scrofulaceum + M. flavescens*	1	(2.2)		1	(2.2)				
* M. simiae + M. cosmeticum*	1	(2.2)		1	(2.2)				
NTM species + MTB	5	(10.9)	1	4	(8.7)	1	1	(2.2)	0
* *MAC + nontypeable NTM + MTB	1	(2.2)		1	(2.2)				
* *Nontypeable NTM + MTB	2	(4.3)		2	(4.3)				
* M. fortuitum* + MTB	1	(2.2)	1	1	(2.2)	1			
* *Unidentified NTM + MTB	1	(2.2)					1	(2.2)	
NTM species unidentified	2	(4.3)	0	2	(4.3)	0	0	(0.0)	0
Total	46	(100.0)	18	33	(69.6)	17	13	(28.3)	1

Abbreviations: MAC, *Mycobacterium avium* complex; MTB, *Mycobacterium tuberculosis*; NTM, nontuberculous mycobacteria.

^a^All NTM patients: including NTM + MTB patients.

^b^Nontypeable NTM: after molecular biology assays, NTM remained nontypeable.

^c^≥2 isolates for MAC.

^d^≥2 isolates for MAC and ≥2 isolates for *M. scrofulaceum.*

^e^Unidentified NTM: identification not performed.

A similar proportion of children with and without NTM isolated initiated anti-TB treatment. Additionally, 12 (26.1%) patients with NTM isolated received specific NTM treatment, initiated at a median time of 6.0 (interquartile range, 2.2–9.3) weeks following inclusion. After 6 months of follow-up, 7 (15.2%) children with NTM had died and 39 were still followed up. Mortality did not significantly differ between children with NTM isolated (7/46 [15.2%]) and the overall cohort (50/381 [13.1%]; *P* = .69).

In multivariate analysis, factors independently associated with NTM isolation were Southeast Asian origin (odds ratio [OR], 7.0; 95% confidence interval [CI], 2.7–18.3), age 5–9 years (OR, 7.7; 95% CI, 1.7–33.9), and severe immunodeficiency (OR, 2.6; 95% CI, 1.2–5.4) (Supplementary Table 2). In Southeast Asian children, age 5–9 years (OR, 7.2; 95% CI, 1.5–35.0), absolute CD4 count <50 cells/µL (OR, 2.6; 95% CI, 1.2–5.7), indeterminate Quantiferon (OR, 11.4; 95% CI, 1.2–108.2), and presence of a BCG scar (OR, 3.0; 95% CI, 1.2–7.2) were independently associated with NTM isolation.

Complementary clinical and biological data on NTM isolation, factors associated with NTM, and comparison of characteristics of children with NTM and those with confirmed TB and NTM positivity rate according to the specimen type are presented in Supplementary Tables 3–5.

## DISCUSSION

In this multicenter study, NTM was isolated in 10.8% of HIV-infected children with suspected TB. Patients with NTM isolates were mostly Southeast Asian children with severe immunodeficiency. MAC was the most frequently identified NTM. To our knowledge, this is the first prospective study specifically investigating the frequency of NTM isolation and associated factors in HIV-infected children with suspected TB in both Africa and Southeast Asia.

The observed higher NTM isolation rate in Southeast Asian as compared to African children may be explained by differential virulence of NTM strains, variable exposure to NTM, and, possibly, genetic factors influencing host susceptibility [4]. High NTM isolation rates were previously reported in Vietnam, Thailand, and Cambodia in HIV-infected individuals >7 years old assessed for pulmonary TB [5, 7]. Higher age associated with NTM isolation in our study is consistent with known bacteriological confirmation challenges in young children, and possibly longer environmental exposure in older children. The rate of NTM isolation in African settings is in line with most previous studies, which reported rates ranging from 2.1% up to 26.3% [8–10]. We identified 11 different NTM species, most of them previously described as pathogenic in HIV-infected patients, except for *Mycobacterium interjectum* and *Mycobacterium cosmeticum* [1, 9]. Among them, MAC, *Mycobacterium kansasii*, and *M. fortuitum* are well-known to be associated with respiratory disease in children [11]. In our study, the most frequently observed species were MAC, mostly in Southeast Asian sites, followed by *M. fortuitum*. Interestingly, *M*. *simiae*, previously associated with disseminated disease and severe outcome in HIV-infected individuals, was identified in 5 Asian children, confirming the circulation of this rarely observed species in this continent [5].

Isolating NTM in respiratory samples of symptomatic individuals is not sufficient to confirm NTM respiratory disease. ATS criteria for NTM diagnosis and treatment decision in adults have not been validated for children, especially HIV-infected children with severe immunodeficiency, and there is currently no internationally accepted definition for NTM respiratory disease in children [2]. In children with severe immunodeficiency, NTM isolation was more frequent, MAC being isolated in more than half of cases, and frequently confirmed on another sample, supporting the hypothesis of NTM-related opportunistic infection in children from our study. However, unlike bacteriologically confirmed TB, which was associated with a 5-fold increase in mortality in ART-naive children in our study despite access to anti-TB treatment, NTM or MAC isolation was not associated with a higher risk of death in children with severe immunodeficiency [12]. In children without severe immunodeficiency, NTM isolation was less frequent, MAC were rare, and only one child had the same species confirmed on a second sample, which is more in favor of environmental exposure and colonization.

Our study had limitations. First, blood cultures were not performed and we may thus have missed NTM disseminated diseases. Second, respiratory samples were not repeated along time, probably contributing to lower confirmation of NTM disease on a second sample. Third, children were managed heterogeneously across sites in the absence of specific recommendations on NTM treatment.

In this international cohort, the rate of NTM isolation in HIV-infected children with suspected TB was high, particularly in Southeast Asian settings. The high proportion of MAC isolated on repeated samples in children with severe immunodeficiency raises the question of genuine respiratory NTM opportunistic infections, even if we did not show a difference in mortality between children with NTM isolated and other children. Further research should be conducted in settings of high TB and NTM burden to assess clinical significance of NTM isolation in HIV-infected children and develop guidelines on NTM pulmonary disease diagnosis and treatment for this population.

## Supplementary Data

Supplementary materials are available at *Clinical Infectious Diseases* online. Consisting of data provided by the authors to benefit the reader, the posted materials are not copyedited and are the sole responsibility of the authors, so questions or comments should be addressed to the corresponding author.

Supplementary Material 1

Supplementary Material

## Notes

***Acknowledgments.*** We thank all children and their parents and caregivers for their participation in the study; National tuberculosis and human immunodeficiency virus programs from participating countries for their support; Françoise Barré-Sinoussi and Jean-François Delfraissy for their continuous support; all the medical, laboratory, and methodology staff for their contribution to the study conduction; the Scientific Committee members for their advices throughout the PAANTHER 01 study; and Brigitte Bazin, Claire Rekacewicz, and Geraldine Colin from the French National Agency for Research on AIDS and Viral Hepatitis (ANRS) for their support.

***Disclaimer.*** The funding sources had no role in study design, data collection and analysis, or the decision to publish this manuscript. The corresponding author had full access to all the data in the study and had final responsibility for the decision to submit for publication.

***Financial support.*** This work was supported by the French National Institute for Health and Medical Research (Inserm) – ANRS (France REcherche Nord&sud Sida-hiv Hépatites) (grant numbers ANRS 12194, ANRS 12229] and Fondation Total.

***Potential conflicts of interest.*** All authors: No reported conflicts. All authors have submitted the ICMJE Form for Disclosure of Potential Conflicts of Interest. Conflicts that the editors consider relevant to the content of the manuscript have been disclosed.
