# Supplementary material for: Isolation of Nontuberculous Mycobacteria in Southeast Asian and African Human Immunodeficiency Virus–infected Children With Suspected Tuberculosis
Source: Clin Infect Dis. 2019 Jan 28;68(10):1750–3. doi: 10.1093/cid/ciy897 (PMC6495014; doi:10.1093/cid/ciy897)
Supplement: Supplementary Material 1 [file ciy897_suppl_supplementary_material-1.docx]

**Supplementary material 1**

**Isolation of Non-tuberculous Mycobacteria in South-East Asian and African HIV-infected Children with Suspected Tuberculosis**

[Supplementary Methods 2](#_Toc526319985)

[Supplementary Results 3](#_Toc526319986)

[Supplementary figure 1. Study flow-chart 4](#_Toc526319987)

[Supplementary table 1. Patients characteristics 5](#_Toc526319988)

[Supplementary table 2. Factors associated with NTM isolation 8](#_Toc526319989)

[Supplementary table 3. Characteristics of children with NTM isolated and children with confirmed TB (excluding those with NTM and MTB positive culture) 10](#_Toc526319990)

[Supplementary table 4: Details on the 46 patients with NTM isolation 12](#_Toc526319991)

[Supplementary table 5: NTM positivity rate according to specimen type. 17](#_Toc526319992)

[Supplementary references 17](#_Toc526319993)

# Supplementary Methods

Study sites

Burkina Faso

Centre Hospitalier Universitaire Souro Sanou, Pediatric Department, Bobo Dioulasso

Cambodia

National Pediatric Hospital, Phnom Penh, Cambodia

Angkor Hospital for Children, Siem Reap, Cambodia

Cameroon

Centre Hospitalier de la Caisse d'Essos, Yaounde

Centre Mère et Enfant de la Fondation Chantal Biya, Yaounde

Vietnam

Pham Ngoc Thach Hospital, Pediatric Department, Ho Chi Minh City

Pediatric Hospital No. 1 (Nhi Dong 1), Infectious Diseases Department, Ho Chi Minh City

Pediatric Hospital No. 2 (Nhi Dong 2), Infectious Diseases Department, Ho Chi Minh City

Ethics committees, institutional review boards, and national authorities

Burkina Faso

Ethics Committee for Research in Health

Cambodia

National Ethics Comity for Health and Research, Phnom Penh, Cambodia

Cameroon

National Ethics Committee

Ministry of Public Health, Division of Health Operations Research

Vietnam

Pham Ngoc Thach Hospital Institutional Review Board

Ho Chi Minh City Department of Health

Ho Chi Minh City People’s Committee

Chest X ray and immunodeficiency methods

CXR were interpreted by an external expert pediatric pulmonologist blinded to clinical and biological data. We used the WHO immunological classification to categorize immunodeficiency as severe according to the age of the child [1].

# Supplementary Results

Children with severe immunodeficiency

NTM were isolated in 33 (13.5%) of 245 children with severe immunodeficiency. Among those, MAC was isolated in more than half, followed by *M. simiae*, and *M. fortuitum*. The same NTM species was identified in at least two separate specimens in 17 children, including 13 (76.5%) with MAC isolated (Table 1). Of 10 children who initiated specific NTM treatment, 8 were still on treatment at the end of the study and 2 had died. Mortality did not differ significantly between those with NTM isolated (7/33; 21.2%) and other children with severe immunodeficiency (40/212; 18.9%; p=0.75), nor did it differ between those with MAC isolated (5/18: 27.8%) and severely immunodeficient children without MAC (42/227; 15.5%; p=0.35).

Children without severe immunodeficiency

NTM were isolated from 13 (7.7%) of the 169 children with no or mild/moderate immunodeficiency. In this group, children with NTM were almost all from Cambodia, (11/13; 84.6%). MAC was identified in 3 (23.1%), and *M. fortuitum* in 5 (38.5%) children. Only one child had an identical species identified on two separate samples (*M. fortuitum)*. Two children received specific NTM treatment. Mortality did not differ between those with NTM isolated (0/13; 0%) and those without NTM (9/156; 5.8%); p=1.0).

Factors associated to MAC isolation

Independent factors positively associated with MAC isolation in South-East Asian children were a CD4 count <50 cells/µl (10.0; 95%CI 3.3-30.4), presence of a BCG scar (5.5; 95%CI 1.4-21.9), elevated AST >2.5xULN (4.5; 95%CI 1.3-16.3), whereas the presence of alveolar opacities on CXR was negatively associated (0.3; 95%CI 0.1-0.9).

Characteristics of patients with NTM and confirmed tuberculosis

Compared to children with confirmed tuberculosis (Supplementary table 3), children with sole NTM isolation had lower rates of BCG immunization, lower median weight for age Z score, lower rates of reported weight loss in the previous 4 weeks, and an abdominal mass more often reported on physical examination. Their median CD4 percentage was lower and they had more frequently elevated LDH >2 ULN and hypereosinophilia (>0.5G/l). They less frequently had anemia, positive QFT results, and positive Acid Fast Bacilli on smear microscopy.

# Supplementary figure 1. Study flow-chart


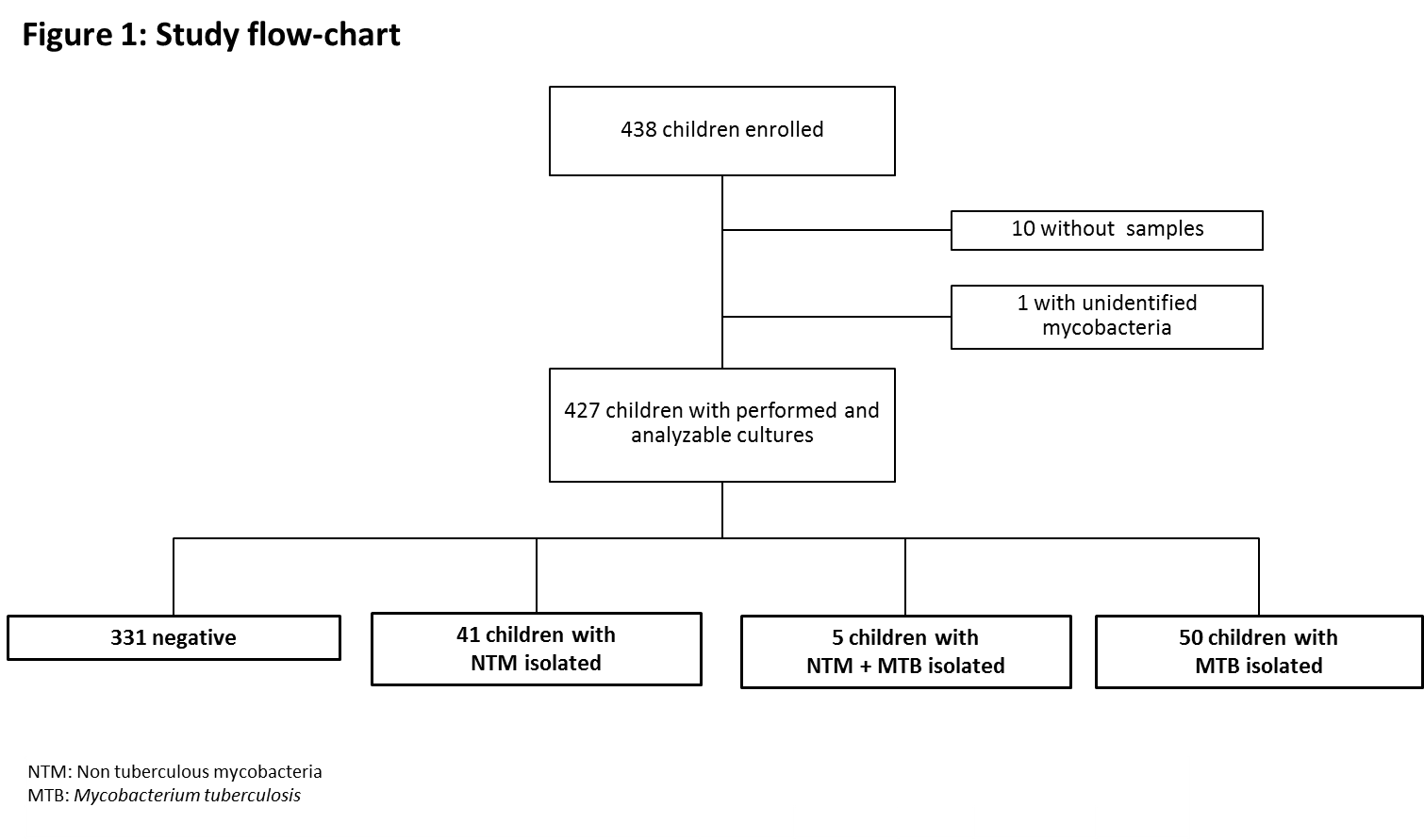


# Supplementary table 1. Patients characteristics

|  | **All**  **(N=427)** | |  | **NTM isolated**  **(N=46)** | |  | **Other children**  **(N=381)** | | ***P-value*** |  |
| --- | --- | --- | --- | --- | --- | --- | --- | --- | --- | --- |
|  | **N*** | **n(%) or**  **median (IQR)** |  | **N*** | **n(%) or median (IQR)** |  | **N*** | **n(%) or median (IQR)** |  |  |
| Country |  |  |  |  |  |  |  |  | *<0,001* |  |
| Burkina Faso |  | 62 (14.5) |  |  | 2 (4.3) |  |  | 60 (15.7) |  |  |
| Cambodia |  | 138 (32.3) |  |  | 21 (45.7) |  |  | 117 (30.7) |  |  |
| Cameroon |  | 120 (28.1) |  |  | 3 (6.5) |  |  | 117 (30.7) |  |  |
| Vietnam |  | 107 (25.1) |  |  | 20 (43.5) |  |  | 87 (22.8) |  |  |
| Sex (male) |  | 212 (49.7) |  |  | 28 (60.9) |  |  | 184 (48.3) | *0.11* |  |
| Age (years) |  | 7.3 [3,4-9,7] |  |  | 7.1 [5,5-9,4] |  |  | 7.3 [3,1-9,8] | *0.28* |  |
| Age categories (years) |  |  |  |  |  |  |  |  | *0.01* |  |
| [0 - 2[ |  | 74 (17.3) |  |  | 2 (4.3) |  |  | 72 (18.9) |  |  |
| [2 - 5[ |  | 64 (15.0) |  |  | 5 (10.9) |  |  | 59 (15.5) |  |  |
| [5 - 10[ |  | 194 (45.4) |  |  | 31 (67.4) |  |  | 163 (42.8) |  |  |
| ≥10 |  | 95 (22.3) |  |  | 8 (17.4) |  |  | 87 (22.8) |  |  |
| Weight for age (z score) | 418 | -2.5 [-3,3 to -1,8] |  |  | -2,7 [-3,7 to -2,0] |  | 372 | -2,5 [-3,2 to -1,7] | *0.04* |  |
| BCG vaccination | 382 | 328 (85.9) |  | 42 | 36 (85.7) |  | 340 | 292 (85.9) | *0.98* |  |
| BCG scar | 424 | 271 (64.1) |  |  | 32 (69.6) |  | 378 | 239 (63.2) | *0.40* |  |
| Tuberculosis contact | 336 | 104 (31.0) |  | 40 | 15 (37.5) |  | 296 | 89 (30.1) | *0.34* |  |
| Tuberculosis history | 408 | 57 (14.0) |  | 44 | 9 (20.5) |  | 364 | 48 (13.2) | *0.19* |  |
| Severe recurrent bacterial pneumonia - WHO | 426 | 39 (9.2) |  |  | 2 (4.3) |  | 380 | 37 (9.7) | *0.29* |  |
| Night sweats | 425 | 154 (36.2) |  |  | 20 (43.5) |  | 379 | 134 (35.4) | *0.28* |  |
| Hemoptysis | 425 | 17 (4.0) |  |  | 3 (6.5) |  | 379 | 14 (3.7) | *0.41* |  |
| Asthenia > 2 w | 423 | 150 (35.5) |  |  | 14 (30.4) |  | 377 | 136 (36.1) | *0.45* |  |
| Fever > 2 w | 419 | 225 (53.7) |  | 45 | 23 (51.1) |  | 374 | 202 (54.0) | *0.71* |  |
| Cough > 2 w | 426 | 356 (83.6) |  |  | 38 (82.6) |  | 380 | 318 (83.7) | *0.85* |  |
| Loss of appetite > 2 w | 393 | 90 (22.9) |  |  | 16 (34.8) |  | 347 | 74 (21.3) | *0.04* |  |
| Digestive signs ⚕ > 2 w | 426 | 100 (23.5) |  |  | 13 (28.3) |  | 380 | 87 (22.9) | *0.42* |  |
| Lymphadenopathy > 1 cm |  | 145 (34.0) |  |  | 15 (32.6) |  |  | 130 (34.1) | *0.84* |  |
| Hepatomegaly | 418 | 144 (34.4) |  | 45 | 21 (46.7) |  | 373 | 123 (33.0) | *0.07* |  |
| Splenomegaly | 420 | 72 (17.1) |  | 45 | 9 (20.0) |  | 375 | 63 (16.8) | *0.59* |  |
| CD4 percentage | 414 | 13,9 [3,2-24] |  |  | 3.7 [0,6-16,2] |  | 368 | 14.6 [4-24,3] | *<0,001* |  |
| CD4 absolute count | 414 | 476 [53-1009] |  |  | 54 [4-745] |  | 368 | 512 [80-1040] | *<0,001* |  |
| Undetectable HIV RNA |  | 101 (23.7) |  |  | 12 (26.1) |  |  | 89 (23.4) | *0.71* |  |
| HIV RNA log10 cp/ml |  | 5,4 [2,6-6,1] |  |  | 5.5 [1,3-6,1] |  |  | 5.2 [2,6-6,1] | *0.87* |  |
| Hemoglobin (g/dl) | 425 | 10,1 [8,6-11,5] |  |  | 10.2 [8,6-11,1] |  | 379 | 10.1 [8,5-11,5] | *0.99* |  |
| Platelets (g/l) | 425 | 327 [245-417] |  |  | 317 [209-388] |  | 379 | 327 [247-426] | *0.18* |  |
| AST (UI/l) | 424 | 42 [31-63] |  |  | 43.5 [31-89] |  | 378 | 42 [31-61] | *0.37* |  |
| ALT (UI/l) | 424 | 29 [19-49] |  |  | 26 [19-49] |  | 378 | 29 [19-49] | *0.46* |  |
| LDH (UI/l) | 378 | 446 [305-801] |  | 38 | 372 [265-599] |  | 340 | 461 [313-825] | *0.02* |  |
| Quantiferon gold in tube ® | 404 |  |  | 41 |  |  | 363 |  | *0.23* |  |
| Positive |  | 49 (12.1) |  |  | 2 (4.9) |  |  | 47 (12.9) |  |  |
| Negative |  | 265 (65.6) |  |  | 27 (65.9) |  |  | 238 (65.6) |  |  |
| Indeterminated |  | 90 (22.3) |  |  | 12 (29.3) |  |  | 78 (21.5) |  |  |
| AFB positive on smear microscopy |  | 29 (6.8) |  |  | 5 (10.9) |  |  | 24 (6.3) | *0.22* |  |
| Positive MTB culture or Xpert MTB/RIF |  | 55 (12.9) |  |  | 5 (10.9) |  |  | 50 (13.1) | *0.67* |  |
| Chest radiograph consistent with TB | 418 | 254 (60.8) |  |  | 27 (58.7) |  | 372 | 227 (61.0) | *0.76* |  |
| Gohn complex on CXR | 418 | 3 (0.7) |  |  | 0 (0.0) |  | 372 | 3 (0.8) | *1.00* |  |
| Excavation on CXR | 418 | 7 (1.7) |  |  | 0 (0.0) |  | 372 | 7 (1.9) | *1.00* |  |
| Alveolar opacity on CXR | 418 | 205 (49.0) |  |  | 19 (41.3) |  | 372 | 186 (50.0) | *0.32* |  |
| Miliary feature on CXR | 417 | 23 (5.5) |  |  | 2 (4.3) |  | 371 | 21 (5.7) | *1.00* |  |
| Nodular infiltration on CXR | 427 | 44 (10.6) |  |  | 6 (13.0) |  | 381 | 38 (10.2) | *0.61* |  |
| Lymphadenopathy or airway compression on CXR ˮ | 418 | 174 (41.6) |  |  | 18 (39.1) |  | 372 | 156 (41.9) | *0.54* |  |
| Pleural effusion on CXR | 418 | 15 (3.6) |  |  | 1 (2.2) |  | 372 | 14 (3.8) | *1.00* |  |
| ART at inclusion | 425 | 169 (39.8) |  |  | 12 (26.1) |  | 379 | 157 (41.4) | *0.06* |  |
| ART duration (months) | 168 | 32 [10-62] |  | 12 | 33 [3-59] |  | 156 | 32 [11-64] | *0.80* |  |
| Initiated tuberculosis treatment |  | 207 (48.5) |  |  | 22 (47.8) |  |  | 185 (48.6) | *0.93* |  |
| Outcome at 6 months |  |  |  |  |  |  |  |  |  |  |
| Death |  | 57 (13.3) |  |  | 7 (15.2) |  |  | 50 (13.1) | *0.69* |  |
| Withdrawal |  | 2 (0.5) |  |  | 1 (2.2) |  |  | 1 (0.3) | *0.20* |  |
| Lost to follow up |  | 6 (1.4) |  |  | 0 (0.0) |  |  | 6 (1.6) | *1.00* |  |

* if n different from N. ⚕ Digestive signs: diarrhea, vomiting, pain > 2 weeks. ˮ Lymphadenopathy or airway compression: peri bronchial or paratracheal adenopathy and/or bronchial and/or tracheal compression. NTM: non tuberculous mycobacteria, MTB: *M. tuberculosis*, AFB: Acid Fast Bacilli, ART: Antiretroviral Treatment

# Supplementary table 2. Factors associated with NTM isolation

|  | Univariate analysis | | | | | | | |  | | Multivariate analysis | | | | | | | | | | | | | | | |
| --- | --- | --- | --- | --- | --- | --- | --- | --- | --- | --- | --- | --- | --- | --- | --- | --- | --- | --- | --- | --- | --- | --- | --- | --- | --- | --- |
|  | OR |  | 95%CI | | |  | *P value* | | |  | | OR | | 95%CI | | | | | | | | | | *P value* | | |
| Asian origin | 7.12 | ( | 2.75 | - | 18.40 | ) | *<0,001* | | |  | | 6.97 | | ( | | 2.65 | | - | | 18.34 | | ) | | *<0,001* | |  |
| Sex (male) | 0.60 | ( | 0.32 | - | 1.12 | ) | *0.11* | | |  | |  | |  | |  | |  | |  | |  | |  | |  |
| Age (years) |  |  |  |  |  |  | *0.01* | | |  | |  | |  | |  | |  | |  | |  | | *0.02* | |  |
| ]2 | 1.00 |  |  |  |  |  |  | | |  | | 1.00 | |  | |  | |  | |  | |  | |  | |  |
| [2 - 5[ | 3.05 | ( | 0.57 | - | 16.30 | ) |  | | |  | | 3.22 | | ( | | 0.58 | | - | | 17.82 | | ) | | | |  |
| [5 - 10[ | 6.85 | ( | 1.60 | - | 29.38 | ) |  | | |  | | 7.67 | | ( | | 1.74 | | - | | 33.86 | | ) | | | |  |
| [10 | 3.31 | ( | 0.68 | - | 16.08 | ) |  | | |  | | 4.60 | | ( | | 0.91 | | - | | 23.21 | | ) | | | |  |
| Weight for age (z score categories) |  |  |  |  |  |  | *0.15* | | |  | |  | |  | |  | |  | |  | |  | |  | |  |
| normal | 1.00 |  |  |  |  |  |  | | |  | |  | |  | |  | |  | |  | |  | |  | |  |
| Moderately underweight | 1.36 | ( | 0.61 | - | 3.01 | ) |  | | |  | |  | |  | |  | |  | |  | |  | |  | |  |
| Severely underweight | 2.10 | ( | 0.98 | - | 4.51 | ) |  | | |  | |  | |  | |  | |  | |  | |  | |  | |  |
| Tuberculosis history | 1.69 | ( | 0.77 | - | 3.74 | ) | *0.19* | | |  | |  | |  | |  | |  | |  | |  | |  | |  |
| Night sweats | 1.41 | ( | 0.76 | - | 2.61 | ) | *0.28* | | |  | |  | |  | |  | |  | |  | |  | |  | |  |
| Hemoptysis | 1.82 | ( | 0.50 | - | 6.58 | ) | *0.36* | | |  | |  | |  | |  | |  | |  | |  | |  | |  |
| Asthenia > 2 w | 1.29 | ( | 0.67 | - | 2.50 | ) | *0.45* | | |  | |  | |  | |  | |  | |  | |  | |  | |  |
| Loss of appetite > 2 w | 1.97 | ( | 1.02 | - | 3.80 | ) | *0.04* | | |  | |  | |  | |  | |  | |  | |  | |  | |  |
| Digestive signs ⚕ > 2 w | 1.33 | ( | 0.67 | - | 2.63 | ) | *0.42* | | |  | |  | |  | |  | |  | |  | |  | |  | |  |
| Respiratory signs > 2 w | 1.03 | ( | 0.56 | - | 1.91 | ) | *0.92* | | |  | |  | |  | |  | |  | |  | |  | |  | |  |
| Cough > 4 w | 1.18 | ( | 0.64 | - | 2.18 | ) | *0.60* | | |  | |  | |  | |  | |  | |  | |  | |  | |  |
| Fever > 4 w | 0.82 | ( | 0.39 | - | 1.71 | ) | *0.59* | | |  | |  | |  | |  | |  | |  | |  | |  | |  |
| Lymphadenopathy > 1 cm | 0.93 | ( | 0.49 | - | 1.79 | ) | *0.84* | | |  | |  | |  | |  | |  | |  | |  | |  | |  |
| Hepatomegaly | 1.78 | ( | 0.95 | - | 3.32 | ) | *0.07* | | |  | |  | |  | |  | |  | |  | |  | |  | |  |
| Splenomegaly | 1.24 | ( | 0.57 | - | 2.70 | ) | *0.59* | | |  | |  | |  | |  | |  | |  | |  | |  | |  |
| Hemoglobin (g/dl) |  |  |  |  |  |  | *0.94* | | |  | |  | |  | |  | |  | |  | |  | |  | |  |
| ]6.5 | 1.00 |  |  |  |  |  |  | | |  | |  | |  | |  | |  | |  | |  | |  | |  |
| [6.5- 9[ | 1.26 | ( | 0.26 | - | 6.05 | ) |  | | |  | |  | |  | |  | |  | |  | |  | |  | |  |
| [9 | 1.30 | ( | 0.29 | - | 5.81 | ) |  | | |  | |  | |  | |  | |  | |  | |  | |  | |  |
| Platelets < 150 G/mm3 | 0.70 | ( | 0.28 | - | 1.76 | ) | *0.45* | | |  | |  | |  | |  | |  | |  | |  | |  | |  |
| ALT > 2.5 ULN (UI/l) | 2.08 | ( | 0.86 | - | 5.05 | ) | *0.11* | | |  | |  | |  | |  | |  | |  | |  | |  | |  |
| LDH (UI/l) |  |  |  |  |  |  | *0.14* | | |  | |  | |  | |  | |  | |  | |  | |  | |  |
| ]2 ULN | 1.00 |  |  |  |  |  |  | | |  | |  | |  | |  | |  | |  | |  | |  | |  |
| [2 ULN -5 ULN[ | 0.59 | ( | 0.19 | - | 1.84 | ) |  | | |  | |  | |  | |  | |  | |  | |  | |  | |  |
| [5 ULN | 0.20 | ( | 0.03 | - | 1.18 | ) |  | | |  | |  | |  | |  | |  | |  | |  | |  | |  |
| missing values | 0.98 | ( | 0.26 | - | 3.63 | ) |  | | |  | |  | |  | |  | |  | |  | |  | |  | |  |
| Immunodeficiency* |  |  |  |  |  |  | *0.13* | | |  | |  | |  | |  | |  | |  | |  | | *0.03* | |  |
| none or not significant | 1.00 |  |  |  |  |  |  | | |  | | 1.00 | |  | |  | |  | |  | |  | |  | |  |
| mild or advanced | 0.57 | ( | 0.12 | - | 2.66 | ) |  | | |  | | 0.81 | | ( | | 0.16 | | - | | 4.06 | | ) | | | |  |
| severe | 1.67 | ( | 0.81 | - | 3.43 | ) |  | | |  | | 2.55 | | ( | | 1.20 | | - | | 5.44 | | ) | | | |  |
| AFB positive on smear microscopy | 1.81 | ( | 0.66 | - | 5.01 | ) | *0.25* | | |  | |  | |  | |  | |  | |  | |  | |  | |  |
| Alveolar opacity on CXR | 1.37 | ( | 0.73 - | | 2.56 | ) | | *0.33* | | |  | |  | |  | |  | |  | |  | |  | |  |  |
| Nodular infiltration on CXR | 0.76 | ( | 0.30 - | | 1.91 | ) | | *0.56* | | |  | |  | |  | |  | |  | |  | |  | |  |  |

NTM: non tuberculous mycobacteria

*Immunodeficiency: defined using the WHO Immunological classification,

⚕ Digestive signs: diarrhea, vomiting, pain > 2 weeks, AFB: Acid Fast Bacilli.

#

# Supplementary table 3. Characteristics of children with NTM isolated and children with confirmed TB (excluding those with NTM and MTB positive culture)

| \|  \| NTM isolated  (N=41) \| \|  \|  \| Confirmed TB  (N=50) \| *P value* \| \| --- \| --- \| --- \| --- \| --- \| --- \| --- \| \|  \| N* \| n(%) or median (IQR) \|  \| N* \| n(%) or median (IQR) \|  \| \| Country \|  \|  \|  \|  \|  \| ***<0.001*** \| \| Burkina Faso \|  \| 1 (2.4) \|  \|  \| 11 (22.0) \|  \| \| Cambodia \|  \| 21 (51.2) \|  \|  \| 4 (8.0) \|  \| \| Cameroon \|  \| 3 (7.3) \|  \|  \| 21 (42.0) \|  \| \| Vietnam \|  \| 16 (39.0) \|  \|  \| 14 (28.0) \|  \| \| Sex (male) \|  \| 25 (61.0) \|  \|  \| 26 (52.0) \| *0.39* \| \| Age (years) \|  \| 8 [6.0-9.5] \|  \|  \| 7.7 [5.6-10.0] \| *0.86* \| \| Weight for age (Z score) \|  \| -2.8 [-3.7 to -2.0] \|  \| 48 \| -2.2 [-3.3 to -1.3] \| ***0.02*** \| \| BCG vaccination \| 38 \| 32 (84.2) \|  \| 45 \| 44 (97.8) \| ***0.04*** \| \| Tuberculosis contact \| 36 \| 14 (38.9) \|  \| 37 \| 16 (43.2) \| *0.71* \| \| Tuberculosis \| 39 \| 9 (23.1) \|  \| 49 \| 5 (10.2) \| *0.10* \| \| Severe recurrent bacterial pneumonia - WHO \|  \| 1 (2.4) \|  \| 49 \| 3 (6.1) \| *0.62* \| \| Night sweats \|  \| 18 (43.9) \|  \| 49 \| 12 (24.5) \| *0.05* \| \| Hemoptysis \|  \| 3 (7.3) \|  \| 49 \| 1 (2.0) \| *0.33* \| \| Weight loss \|  \| 25 (61.0) \|  \| 49 \| 43 (87.8) \| ***<0.01*** \| \| Asthenia > 2 w \|  \| 14 (34.1) \|  \| 48 \| 25 (52.1) \| *0.09* \| \| Fever > 2 w \| 40 \| 19 (47.5) \|  \| 47 \| 31 (66.0) \| *0.08* \| \| Cough > 2 w \|  \| 34 (82.9) \|  \| 49 \| 40 (81.6) \| *0.87* \| \| Loss of appetite > 2 w \|  \| 15 (36.6) \|  \| 40 \| 7 (17.5) \| *0.05* \| \| Digestive signs ⚕ > 2 w \|  \| 12 (29.3) \|  \| 49 \| 15 (30.6) \| *0.89* \| \| Lymphadenopathy > 1 cm \|  \| 11 (26.8) \|  \|  \| 17 (34.0) \| *0.46* \| \| Hepatomegaly \| 40 \| 16 (40.0) \|  \| 48 \| 19 (39.6) \| *0.97* \| \| Splenomegaly \| 40 \| 6 (15.0) \|  \| 48 \| 16 (33.3) \| *0.05* \| \| Abdominal mass \|  \| 0 (0.0) \|  \| 49 \| 6 (12.2) \| ***0.03*** \| \| CD4 percentage \|  \| 3.2 [1.0-19.0] \|  \|  \| 10.5 [3.4-25.0] \| ***0.01*** \| \| Immunodeficiency - WHO \|  \|  \|  \|  \|  \| *0.86* \| \| not significant \|  \| 10 (24.4) \|  \|  \| 15 (30.0) \|  \| \| mild to advanced \|  \| 2 (4.9) \|  \|  \| 2 (4.0) \|  \| \| severe \|  \| 29 (70.7) \|  \|  \| 33 (66.0) \|  \| \| HIV RNA log10 cp/ml \|  \| 5.4 [1.3-6.1] \|  \|  \| 5.8 [4.3-6.5] \| *0.06* \| \| Undetectable HIV RNA \|  \| 11 (26.8) \|  \|  \| 4 (8.0) \| ***0.02*** \| \| Hemoglobin (g/dl) \|  \| 10.4 (25.4) \|  \|  \| 8.5 [6.7-10.3] \| ***<0.001*** \| \| Platelets (G/l) \|  \| 320 [216-390] \|  \|  \| 294 [231-357] \| *0.78* \| \| Neutrophiles G/l, med [IQR] \|  \| 4 [1.6-5.3] \|  \|  \| 4.3 [3.0-7.7] \| *0.06* \| \| Eosinophiles > 0,5 G/l \|  \| 10 (24.4) \|  \|  \| 2 (4.0) \| ***<0.01*** \| \| AST (UI/L) \|  \| 40 [31-89] \|  \|  \| 45 [32-64] \| *0.95* \| \| ALT (UI/L) \|  \| 22 [19-45] \|  \|  \| 26 [16-51] \| *0.93* \| \| LDH (UI/L) \| 33 \| 350 [265-521] \|  \| 44 \| 678 [396-1379] \| ***<0.001*** \| \| Positive TST \| 39 \| 2 (5.1) \|  \| 41 \| 7 (17.1) \| *0.16* \| \| Quantiferon Gold in Tube ® \| 36 \|  \|  \| 49 \|  \| ***<0.001*** \| \| Positive \|  \| 0 (0.0) \|  \|  \| 22 (44.9) \|  \| \| Negative \|  \| 26 (72.2) \|  \|  \| 14 (28.6) \|  \| \| Indeterminate \|  \| 10 (27.1) \|  \|  \| 13 (26.5) \|  \| \| Positive AFB test \|  \| 4 (9.4) \|  \|  \| 23 (46.0) \| ***<0.001*** \| \| Chest radiograph consistent with TB \|  \| 23 (56.2) \|  \| 46 \| 32 (69.6) \| *0.27* \| \| Excavation on CXR \|  \| 0 (0.0) \|  \| 46 \| 1 (2.2) \| *1.00* \| \| Miliary feature on CXR \|  \| 1 (2.1) \|  \| 46 \| 7 (15.2) \| *0.06* \| \| Lymphadenopathy or airway compression on CXR ˮ \|  \| 14 (34.1) \|  \| 46 \| 20 (43.5) \| *0.39* \| \| ART at inclusion \|  \| 11 (26.1) \|  \| 48 \| 14 (29.2) \| *0.81* \| \| Initiated tuberculosis treatment ** \|  \| 19 (46.1) \|  \|  \| 38 (76.0) \| ***<0.01*** \| \| Death \|  \| 6 (14.6) \|  \|  \| 15 (30.0) \| *0.08* \|  \| Data are n (%) or median [IQR].Treatment. \| \| --- \| \| * if n different from N \| \| NTM: non tuberculous mycobacteria, MTB: M. tuberculosis, AFB: Acid Fast Bacilli, ARV: antiretroviral \| \| ⚕ Digestive signs: diarrhea, vomiting, or pain > 2 weeks \| \| **ˮ** Lymphadenopathy or airway compression on CXR: peri bronchial or paratracheal adenopathy and/or bronchial and/or tracheal compression \| \| ** Initiated tuberculosis treatment: 3 or 4therapy (Rifampicine, Izoniazid, Pyrazinamid +/- Ethambutol) at any time \| |
| --- | --- | --- | --- | --- | --- | --- | --- | --- | --- | --- | --- | --- | --- | --- | --- | --- | --- | --- | --- | --- | --- | --- | --- | --- | --- | --- | --- | --- | --- | --- | --- | --- | --- | --- | --- | --- | --- | --- | --- | --- | --- | --- | --- | --- | --- | --- | --- | --- | --- | --- | --- | --- | --- | --- | --- | --- | --- | --- | --- | --- | --- | --- | --- | --- | --- | --- | --- | --- | --- | --- | --- | --- | --- | --- | --- | --- | --- | --- | --- | --- | --- | --- | --- | --- | --- | --- | --- | --- | --- | --- | --- | --- | --- | --- | --- | --- | --- | --- | --- | --- | --- | --- | --- | --- | --- | --- | --- | --- | --- | --- | --- | --- | --- | --- | --- | --- | --- | --- | --- | --- | --- | --- | --- | --- | --- | --- | --- | --- | --- | --- | --- | --- | --- | --- | --- | --- | --- | --- | --- | --- | --- | --- | --- | --- | --- | --- | --- | --- | --- | --- | --- | --- | --- | --- | --- | --- | --- | --- | --- | --- | --- | --- | --- | --- | --- | --- | --- | --- | --- | --- | --- | --- | --- | --- | --- | --- | --- | --- | --- | --- | --- | --- | --- | --- | --- | --- | --- | --- | --- | --- | --- | --- | --- | --- | --- | --- | --- | --- | --- | --- | --- | --- | --- | --- | --- | --- | --- | --- | --- | --- | --- | --- | --- | --- | --- | --- | --- | --- | --- | --- | --- | --- | --- | --- | --- | --- | --- | --- | --- | --- | --- | --- | --- | --- | --- | --- | --- | --- | --- | --- | --- | --- | --- | --- | --- | --- | --- | --- | --- | --- | --- | --- | --- | --- | --- | --- | --- | --- | --- | --- | --- | --- | --- | --- | --- | --- | --- | --- | --- | --- | --- | --- | --- | --- | --- | --- | --- | --- | --- | --- | --- | --- | --- | --- | --- | --- | --- | --- | --- | --- | --- | --- | --- | --- | --- | --- | --- | --- | --- | --- | --- | --- | --- | --- | --- | --- | --- | --- | --- | --- | --- | --- | --- | --- | --- | --- | --- | --- | --- | --- | --- | --- | --- | --- | --- | --- | --- | --- | --- | --- | --- | --- | --- | --- | --- | --- | --- | --- | --- | --- | --- | --- | --- | --- | --- | --- | --- | --- | --- | --- | --- | --- | --- | --- | --- | --- | --- | --- | --- | --- | --- | --- | --- | --- | --- | --- | --- | --- | --- | --- | --- | --- | --- | --- | --- | --- | --- |

# Supplementary table 4: Details on the 46 patients with NTM isolation

| At Inclusion | | | | | | | | | |  |  | During follow up | | |  | End of study | |
| --- | --- | --- | --- | --- | --- | --- | --- | --- | --- | --- | --- | --- | --- | --- | --- | --- | --- |
| Child | Origin | Age (y) | WAZ | CD4  %/mm^3^ (abs/mm^3^) | Months on ART | HIV RNA (Log10 cp /ml) | Chest X-Ray signs consistent with TB | Isolates | Nb. of isolates | Acid Fast Bacilly |  | TB ttt | NTM ttt (continued or not at the end of study) | Delay to NTM ttt |  | Outcome (delay to death in days) | Weight gain (kg) |
| 1 | CA | 9.3 | -3.0 | 1% (10) |  | 5.4 | alveolar opacities | ***M. avium*** | 5 |  |  |  |  |  |  | alive | 5 |
| 2 | KH | 3.6 | -4.1 | 0% (0) |  | 6.4 | alveolar opacities | ***M. avium*** | 3 |  |  | RHZE | azm + E + R (death) | 13 |  | death (43) |  |
| 3 | KH | 6.4 | -3.4 | 1% (4) |  | 6.9 |  | ***M. avium*** | 5 | Positive |  | RHZE | azm + E + R (death) | 20 |  | death (78) |  |
| 4 | VN | 9.2 | -1.5 | 1% (32) |  | 6.2 | alveolar opacities | ***M. avium*** | 2 |  |  | RHZE | clr + E + FQ (continued) | 66 |  | alive | 1.5 |
| 5 | VN | 6.8 | -3.7 | 1% (4) |  | 5.6 | paratracheal adenopathy | ***M. avium*** | 6 | Positive |  | RHZE | azm + E + R + lvx (continued) | 62 |  | alive | 1 |
| 6 | VN | 11.3 | -2.0 | 0% (3) |  | 5.6 |  | ***M. avium* non typable NTM** | 3 2 |  |  |  |  |  |  | death (16) |  |
| 7 | VN | 4.8 | -2.4 | 2% (34) |  | 5.4 | alveolar opacities, perihilar adenopathy | ***M. avium* non typable NTM** | 2 2 |  |  | RHZE | clr + E + R (continued) | 31 |  | alive | 2.5 |
| 8 | VN | 5.5 | -1.5 | 0% (6) |  | 6.1 |  | ***M. avium* non typable NTM** | 2 2 |  |  |  |  |  |  | death (32) |  |
| 9 | KH | 6.8 | -3.7 | 1% (9) |  | 6.6 | alveolar opacities | ***M. intracellulare*** | 4 |  |  |  | azm + E (continued) | 64 |  | alive | 3 |
| 10 | KH | 11.1 | -2.0 | 9% (198) |  | 5.9 |  | ***M. intracellulare*** | 2 |  |  | RHZE | azm + E + R (continued) | 18 |  | alive | 3 |
| 11 | VN | 8.0 | -3.6 | 2% (39) |  | 5.8 | alveolar opacities | ***M. intracellulare*** | 3 | Positive |  |  | azm + E + R + lvx  (continued) | 11 |  | alive | 3.5 |
| 12 | VN | 9.5 | -4.8 | 1% (4) |  | 6.3 |  | ***M. intracellulare*** | 3 |  |  |  |  |  |  | alive | 4 |
| 13 | VN | 6.1 | -1.5 | 9% (147) |  | 6.3 |  | ***M. intracellulare*** | 1 |  |  |  |  |  |  | alive | 4.5 |
| 14 | KH | 9,4 | -3,0 | 24% (1325) | 56,1 | undetectable | perihilar adenopathy | ***M. intracellulare*** | 1 |  |  |  |  |  |  | alive | 3 |
| 15 | KH | 6,1 | -0,9 | 20% (852) | 5,2 | undetectable |  | ***M. intracellulare*** | 1 |  |  | RHZE | azm + E + R (stopped) | 67 |  | alive | 1,5 |
| 16 | VN | 9,9 | -2,0 | 27% (692) | 44,0 | undetectable |  | ***M. intracellulare*** | 1 |  |  |  |  |  |  | alive | 1,5 |
| 17 | VN | 8.3 | -2.9 | 3% (11) |  | 5.2 | alveolar opacities /pleural effusion | ***M. intracellulare M. simiae*** | 1 1 |  |  |  |  |  |  | alive | 1 |
| 18 | VN | 9.4 | -4.5 | 0% (2) |  | 5.3 | alveolar opacities | ***M. intracellulare M. simiae*** | 1 1 |  |  | RHZE | azm + E + R (continued) | 53 |  | alive | 0.5 |
| 19 | VN | 10.4 | -2.5 | 1% (4) |  | 5.9 |  | ***M. intracellulare M. scrofulaceum*** | 2 3 | Positive |  | RHZE |  |  |  | death (13) |  |
| 20 | VN | 9.2 | -6.0 | 0% (2) |  | 4.5 |  | ***M. intracellulare* non identified NTM** | 1 1 |  |  |  |  |  |  | alive | 7 |
| 21 | VN | 0.3 | -2.1 | 16% (819) | 0.8 | 5.8 | perihilar adenopathy | ***M. intracellulare* non typable NTM MTB** | 1 1 |  |  | RHZE |  |  |  | alive | 1.7 |
| 22 | CA | 7.0 | -5.2 | 1% (8) |  | 5.8 | alveolar opacities | ***M. fortuitum*** | 1 |  |  |  |  |  |  | alive | 5.3 |
| 23 | KH | 2.2 | -4.3 | 12% (1349) | 18 | 5.1 |  | ***M. fortuitum*** | 1 |  |  | RHZE |  |  |  | alive | 1.6 |
| 24 | KH | 11.8 | -2.5 | 6% (85) |  | 5.7 | alveolar opacities | ***M. fortuitum*** | 1 |  |  |  |  |  |  | alive | 3.5 |
| 25 | VN | 2.9 | -4.1 | 0% (2) |  | 5.5 | alveolar opacities, nodular infiltrations | ***M. fortuitum* MTB** | 4 |  |  | RHZE |  |  |  | alive |  |
| 26 | KH | 5,5 | -1,8 | 19% (893) |  | 3,1 | alveolar opacities / perihilar + paratracheal adenopathy / tracheal compression | ***M. fortuitum*** | 1 |  |  | RHZE |  |  |  | alive | 3 |
| 27 | KH | 6,8 | -1,8 | 23% (623) |  | undetectable | nodular infiltrations / perihilar adenopathy | ***M. fortuitum*** | 2 |  |  | RHZE |  |  |  | alive | 3 |
| 28 | KH | 6,5 | -3,83 | 15% (428) | 22,9 | 5,8 | alveolar opacities / paratracheal adenopathy | ***M. fortuitum*** | 1 |  |  | RHZE |  |  |  | alive | 1 |
| 29 | KH | 9,0 | -2,1 | 31% (1032) | 60,0 | undetectable |  | ***M. fortuitum*** | 1 |  |  | RHZE |  |  |  | alive | 2,5 |
| 30 | KH | 10,1 | -2,5 | 37% (1073) | 79,6 | undetectable |  | ***M. fortuitum M. interjectum*** | 1 1 |  |  |  |  |  |  | alive | 0,5 |
| 31 | KH | 12,2 | -2,5 | 27% (907) | 91,0 | undetectable | alveolar opacities / nodular infiltrations / perihilar + paratracheal adenopathy | ***M. interjectum*** | 1 |  |  |  |  |  |  | alive | 0,5 |
| 32 | VN | 5.5 | -1.8 | 10% (184) |  | 5.1 | alveolar opacities | ***M. kansasii*** | 2 |  |  |  |  |  |  | alive |  |
| 33 | KH | 7,3 | -1,83 | 26% (858) |  | 5,4 | perihilar + paratracheal adenopathy / tracheal compression | ***M. gordonae*** | 1 |  |  |  |  |  |  | alive | 0,5 |
| 34 | KH | 6,2 | -3,2 | 25% (1433) | 58,8 | undetectable | perihilar + paratracheal adenopathy | ***M. gordonae* non typable NTM** | 1 1 |  |  | RHZE | azm + E (continued) | 95 |  | alive | 1 |
| 35 | KH | 6.1 | -3.3 | 4% (131) | 1 | undetectable | alveolar opacities | ***M. lentiflavum*** | 1 |  |  |  |  |  |  | death (5) |  |
| 36 | KH | 5.3 | -4.6 | 1% (6) |  | 6.1 |  | ***M. scrofulaceum*** | 2 |  |  | RHZE |  |  |  | alive | 3 |
| 37 | KH | 8,0 | -3,56 | 23% (340) | 1,5 | undetectable | alveolar opacities / perihilar adenopathy | ***M. scrofulaceum*** | 1 |  |  | RHZE |  |  |  | alive | 2,5 |
| 38 | VN | 2.6 | -2.1 | 13% (1104) |  | 6.3 | alveolar opacities, miliary, perihilar adenopathy | ***M. scrofulaceum M. flavescens*** | 1 1 |  |  | RHZ |  |  |  | alive | 1.8 |
| 39 | KH | 5.1 | -3.5 | 1% (17) |  | 6.4 | nodular infiltrations, perihilar adenopathy | ***M. simiae*** | 1 |  |  | RHZE |  |  |  | alive | 2.5 |
| 40 | KH | 8.5 | -4.3 | 3% (25) |  | 6.4 | nodular infiltrations | ***M. simiae*** | 2 |  |  | RHZE | azm + E + R (continued) | 7 |  | alive | 4.5 |
| 41 | VN | 10.9 | -2.0 | 0% (2) |  | 5.3 | alveolar opacities, perihilar adenopathy | ***M. simiae M. cosmeticum*** | 1 1 |  |  | RHZE |  |  |  | alive | 3 |
| 42 | CA | 11.9 | -2.5 | 1% (4) |  | 5.2 | alveolar opacities, perihilar adenopathy, bronchial compression | **non identified NTM** | 3 |  |  |  |  |  |  | alive | -0.3 |
| 43 | BF | 9.7 | -2.8 | 10% (227) |  | und. |  | **non identified NTM** | 2 |  |  |  |  |  |  | alive | 0.5 |
| 44 | BF | 6,1 | 2,47 | 16% (745) |  | undetectable | nodular infiltrations /perihilar adenopathy | **non identified NTM**  **MTB** | 2 |  |  | RHZ |  |  |  | alive | 3,5 |
| 45 | VN | 1.8 | -3.9 | 0% (4) |  | 6.2 |  | **non typable NTM MTB** | 1 |  |  | RHZE |  |  |  | death (30) |  |
| 46 | VN | 8.8 | -0.2 | 8% (69) |  | 5.8 | miliary, perihilar adenopathy | **non typable NTM MTB** | 1 | Positive |  | RHZE |  |  |  | alive | -3 |

CA, Cameroon; KH, Cambodia; VN, Vietnam; BF, Burkina Faso; NTM, non-tuberculous mycobacteria; MTB, mycobacterium tuberculosis; TB, Tuberculosis; ART, Antiretroviral treatment; R, Rifampicin; H, Isoniazid; Z, Pyrazinamid; E, Ethambutol; FQ, fluoroquinolone; Lfx, levofloxacine; AZI, azithromycin; clarithro, clarithromycin.

# Supplementary table 5: NTM positivity rate according to specimen type.

|  | Samples collected  N | NTM-positive specimen  n (%) |
| --- | --- | --- |
| Gastric aspirate | 580 | 45 (7.8) |
| Naso Pharyngeal aspirate | 414 | 17 (4.1) |
| Sputum | 230 | 12 (5.2) |
| String Test | 165 | 13 (7.9) |
| Stools | 414 | 15 (3.6) |

# Supplementary references

1. WHO | WHO case definitions of HIV for surveillance and revised clinical staging and immunological classification of HIV-related disease in adults and children. Available at: http://www.who.int/hiv/pub/guidelines/hivstaging/en/. Accessed 30 July 2018.

2. McCarthy KD, Cain KP, Winthrop KL, et al. Nontuberculous mycobacterial disease in patients with HIV in Southeast Asia. Am J Respir Crit Care Med **2012**; 185:981–988.

3. Hatherill M, Hawkridge T, Whitelaw A, et al. Isolation of Non-Tuberculous Mycobacteria in Children Investigated for Pulmonary Tuberculosis. PLoS ONE **2006**; 1:e21.

4. López-Varela E, García-Basteiro AL, Augusto OJ, et al. High Rates of Non-Tuberculous Mycobacteria Isolation in Mozambican Children with Presumptive Tuberculosis. PloS One **2017**; 12:e0169757.
