# Supplementary material for: Isolation of Nontuberculous Mycobacteria in Southeast Asian and African Human Immunodeficiency Virus–infected Children With Suspected Tuberculosis
Source: Clin Infect Dis. 2019 Jan 28;68(10):1750–3. doi: 10.1093/cid/ciy897 (PMC6495014; doi:10.1093/cid/ciy897)
Supplement: Supplementary Material [file ciy897_suppl_supplementary_material-2.docx]

**Supplementary material 2: List of members of the ANRS 12229 PAANTHER study group**

**Isolation of Non-tuberculous Mycobacteria in South-East Asian and African HIV-infected Children with Suspected Tuberculosis**

**Coordination teams:**

Epidemiology and Public Health Unit, Institut Pasteur du Cambodge, Phnom Penh, Cambodia: Sao Sarady Ay, Laurence Borand (Regional Coordinator - Asia), Malen Chan, Phalla Chea, Bunnet Dim, Sophie Goyet, Olivier Marcy (Coordinating Investigator), Nimul Roat Men, Phearavin Pheng, Susan Ramsay, Sophea Suom, Arnaud Tarantola, Naisim Te, Sreymom Than, Sovannary Ung.

Centre Pasteur du Cameroun, Yaoundé, Cameroon, Epidemiology Unit: Verlaine Bolyse Mbouchong, Francine Nanda, Mathurin Tejiokem (Country Principal Investigator).

Institut Recherche et Développement, Montpellier, France: Philippe Msellati (Regional Coordinator - Africa)

Clinical Research Unit, Pham Ngoc Thach Hospital, Ho Chi Minh City, Vietnam: Thi Oanh Nguyen, Thi Ngoc Lan Nguyen, Catherine Quillet, Khanh Lê Quoc, My Huong To, Thi Kim Phung Tran.

Centre Hospitalier Necker – Enfants Malades, Assistance Publique-Hôpitaux de Paris, Paris, France: Stéphane Blanche, Christophe Delacourt, Laureline Berteloot.

Centre Hospitalier Pitié-Salpêtrière, Assistance Publique-Hôpitaux de Paris, Paris, France: Guislaine Carcelain

Centre Hospitalier Arnaud de Villeneuve, Laboratoire de Microbiologie, Montpellier, France: Sylvain Godreuil

Inserm SC10, Villejuif, France: Isabelle Fournier-Nicolle

**Clinical sites and teams:**

Centre Hospitalier Universitaire Souro Sanou, Bobo Dioulasso, Burkina Faso: Boubacar Nacro (Country Principal Investigator), Zakari Nikiema, Diane Ursule Sangare/Ouattara, Bintou Sanogo, Edgar Sib Sié, Seydou Traore.

National Pediatric Hospital, Phnom Penh, Cambodia: Chorn Chhuk, Chantheany Huot, Sang Han Kath, Sokoeun Khin, Peou Kim, Kim Eng Lim, Phary Lim, Vannareth Lim, Yany Nav, Boren Pich, Sotharin Tuy, Vibol Ung (Coordinating Investigator)

Angkor Hospital for Children, Siem Reap, Cambodia: Phal Chea, Sengtray Chhraing, Bophal Chuop, Ramy Hem, Phanoeurn Heng, Seng Hap Huon, Sokhorn Khum, Thear Liv, Vichea Ly, Makara Moch, Leakheana Neou (Site Principal Investigator), Phanith Rey, Vina Sath, Nopheavann Sun.

Centre Hospitalier de la Caisse d'Essos, Yaounde, Cameroon: Minkala Mvogo, Jean Pierre Ndongo, Laurence Ngassam, Patrice Assene Ngo’o, Marcelle Njind Nkoum, Anne Esther Njom Lend, Marie Flore Oyanche, Suzie Tetang Moyo (Site Principal Investigator),

Centre Mère et Enfant de la Fondation Chantal Biya, Yaounde, Cameroon: Francis Ateba Ndongo (Site Principal Investigator), Suzanne Balla, Jean Marie Ehongo Amanya, Paul Koki Ndombo, Elisabeth Mangoya Marie Josée Mapah Kambaing, Sylviane Ndjantou, Jean Audrey Ndongo, Roger Ngoya Abiguide, Angeline Nkembe, Jean-Voisin Taguebue.

Pham Ngoc Thach Hospital, Pediatric Department, Ho Chi Minh City, Vietnam: Chau Giang Do, Duc Bang Nguyen, Ngo Vi Vi Nguyen, Thi Hieu Nguyen, Thi Thanh Thanh Nguyen, Ngoc Duong Tran (Site Principal Investigator), Duy An Vo (radiology).

Pediatric Hospital No. 1, Infectious Diseases Department, Ho Chi Minh City, Vietnam: Thi Xuan Lan Dang, Thi Kim Phuong Do, Thi Thanh Tuyen Dang, Hoang Chau Nguyen, Ngoc Tuong Vy Pham, Huu Khanh Truong (Site Principal Investigator)

Pediatric Hospital No. 2, Infectious Diseases Department, Ho Chi Minh City, Vietnam: Chau Viet Do (Site Principal Investigator), Bich Ngoc Huynh, Thi Thanh Thuy Le, Thi Thu Hiep Nguyen, Ngoc Luu Tran, Thien An Vu.

**Laboratories**

Centre Hospitalier Universitaire Souro Sanou, Bobo Dioulasso, Burkina Faso: Anselme Millogo, Abdoul Salam Ouedraogo.

Centre Muraz Laboratory, Bobo Dioulasso, Burkina Faso: Michel Gomgnimbou, Antoinette Kabore, Moumini Nouctara, Dézémon Zingué.

National Pediatric Hospital, Laboratory, Phnom Penh, Cambodia: Pheron Chat, Viso Srey.

Angkor Hospital for Children, Laboratory, Siem Reap, Cambodia: Sitha Chheang, Putchhat Hor, Manory Oum.

Institut Pasteur du Cambodge, Laboratory, Phnom Penh, Cambodia: Keo Monorea, Kou Sokchea, Cheng Sokleaph, Alexandra Kerleguer.

Centre Pasteur du Cameroun, Microbiology Laboratory, Yaounde, Cameroon: Sara Eyangoh, Afi Leslie Kaiyven, Samuel Walter Kokola Bayanak Sylvie Georgette Zebaze.

Pham Ngoc Thach Hospital, Microbiology Laboratory, Ho Chi Minh City, Vietnam: Huu Loc Tran, Manh Hoang Nguyen, Ngoc Lan Nguyen, Thi Cao Van Nguyen, Thu Hang Pham, Dinh Quyen Phan, Thi Ha Vo.

Pasteur Institute, Virology Department, Ho Chi Minh City, Vietnam: Xuan Lien Truong, Xuan Thinh Vu.

**Scientific Committee**

Stéphane Blanche, Necker Hospital, Paris (Chair); Olivier Marcy, Institut Pasteur in Cambodia, Phnom Penh, Cambodia; Ung Vibol, National Pediatric Hospital, Phnom Penh, Cambodia; Nguyen Thy Ngoc Lan, Pham Ngoc Thach Hospital, Ho Chi Minh City, Vietnam; Mathurin Tejiokem, Centre Pasteur du Cameroun, Yaounde, Cameroon; Boubacar Nacro, Centre Hospitalier Universitaire Souro Sanou, Bobo Dioulasso, Burkina Faso; Arnaud Tarantola, Epidemiologist, Institut Pasteur in Cambodia, Phnom Penh, Cambodia; Christophe Delacourt, Necker Hospital, Paris, France; Guislaine Carcelain, Pitié-Salpêtrière Hospital, Paris, France; Sylvain Godreuil, Centre Hospitalier Arnaud de Villeneuve, Montpellier, France; David AJ Moore, London School of Hygiene and tropical Medicine, London, United Kingdom; Philippe Mselatti, IRD, Montpellier, France; Isabelle Fournier, ANRS, Phnom Penh, Cambodia; Didier Laureillard, ANRS, Ho Chi Minh City, Vietnam; Truong Huu Khann, Nhi Dong 1 Hospital, Ho Chi Minh City, Vietnam; Xavier Blanc, Bicêtre Hospital, Le Kremlin-Bicêtre, France;

Géraldine Colin, Paula Garcia, and Claire Rekacewicz, ANRS, France.
